# The structure of the deubiquitinase USP15 reveals a misaligned catalytic triad and an open ubiquitin-binding channel

**DOI:** 10.1074/jbc.RA118.003857

**Published:** 2018-09-18

**Authors:** Stephanie J. Ward, Hayley E. Gratton, Peni Indrayudha, Camille Michavila, Rishov Mukhopadhyay, Sigrun K. Maurer, Simon G. Caulton, Jonas Emsley, Ingrid Dreveny

**Affiliations:** From the Centre for Biomolecular Sciences, School of Pharmacy, University of Nottingham, Nottingham NG7 2RD, United Kingdom

**Keywords:** crystal structure, deubiquitylation (deubiquitination), cysteine protease, protein conformation, ubiquitin, protease, protein degradation, catalytic triad, ubiquitin-specific protease, USP15

## Abstract

Ubiquitin-specific protease 15 (USP15) regulates important cellular processes, including transforming growth factor β (TGF-β) signaling, mitophagy, mRNA processing, and innate immune responses; however, structural information on USP15's catalytic domain is currently unavailable. Here, we determined crystal structures of the USP15 catalytic core domain, revealing a canonical USP fold, including a finger, palm, and thumb region. Unlike for the structure of paralog USP4, the catalytic triad is in an inactive configuration with the catalytic cysteine ∼10 Å apart from the catalytic histidine. This conformation is atypical, and a similar misaligned catalytic triad has so far been observed only for USP7, although USP15 and USP7 are differently regulated. Moreover, we found that the active-site loops are flexible, resulting in a largely open ubiquitin tail–binding channel. Comparison of the USP15 and USP4 structures points to a possible activation mechanism. Sequence differences between these two USPs mainly map to the S1′ region likely to confer specificity, whereas the S1 ubiquitin–binding pocket is highly conserved. Isothermal titration calorimetry monoubiquitin- and linear diubiquitin-binding experiments showed significant differences in their thermodynamic profiles, with USP15 displaying a lower affinity for monoubiquitin than USP4. Moreover, we report that USP15 is weakly inhibited by the antineoplastic agent mitoxantrone *in vitro*. A USP15–mitoxantrone complex structure disclosed that the anthracenedione interacts with the S1′ binding site. Our results reveal first insights into USP15's catalytic domain structure, conformational changes, differences between paralogs, and small-molecule interactions and establish a framework for cellular probe and inhibitor development.

## Introduction

Ubiquitin-specific proteases (USPs)^5^ are key players in the regulation of important cellular signaling pathways through catalyzing the deconjugation reaction of ubiquitin from substrate proteins (1). Although the dysregulation or malfunction of USPs has been linked to diseases as varied as cancer, neurodegenerative disorders, and a host's response to infection (2, 3), for most of these enzymes, there is a lack of molecular understanding regarding conformational changes, regulation of catalysis, and specificity. Structural information is key to shedding light on their mechanism and substrate interactions and can aid small-molecule inhibitor development. The multifunctional protease USP15 regulates several important pathways in health and disease (4), including TGF-β (5), IGF-I (6), innate immune signaling (7), mRNA processing (8), and mitophagy (9). Furthermore, USP15 can promote new protein synthesis (10), depletion results in DNA double strand repair defects (11), and USP15 was shown to regulate the ligase MDM2 with effects on the stability of p53 in cancer cells and the T-cell transcription factor, NFATc2 (12). USP15 is also dysregulated in many cancers (12–14), and knockdown of USP15 rescues the mitophagy defect of Parkinson's disease patients' fibroblasts (9). USP15 has two paralogs, the more closely related USP4 and more distant USP11, which we refer to as the DUSP–UBL family of USPs (15–18). Viability in mice is contingent on a functional copy of USP4 or USP15 (18), and the two paralogs share a degree of functional overlap in mRNA splicing (8, 19, 20), TGF-β signaling (21), and RIG-I–mediated antiviral signaling (7, 22), albeit mostly not acting on the same substrates in these pathways.

**Figure 1.**
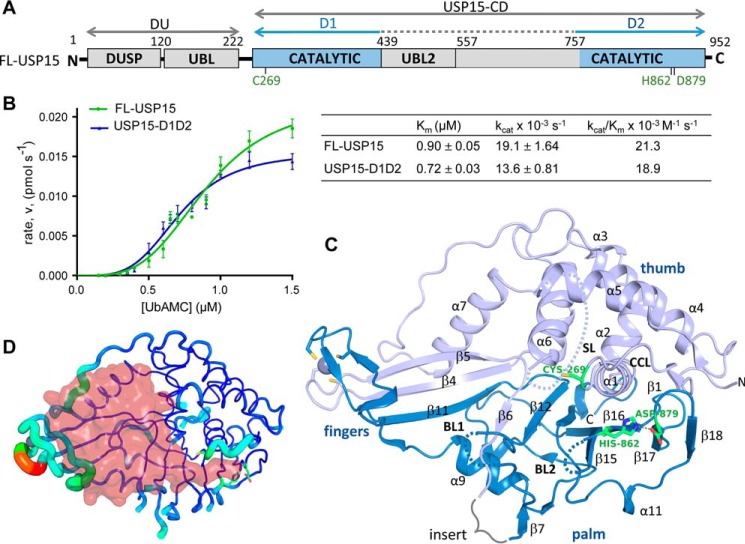
**Kinetic parameters and crystal structure of the USP15 catalytic core.** *A*, *schematic representation* of the human USP15 domain structure highlighting the location of the catalytic core region encompassing the subdomain halves D1 and D2 in *blue* and the catalytic triad residues (*green*) with additional domains *labeled* as *DUSP* (domain present in USPs) and *UBL* (ubiquitin-like). *B*, kinetic assays of FL-USP15 and USP15-D1D2 using ubiquitin-AMC as a substrate with FL-USP15 in *green* and USP15-D1D2 in *blue*. Each point represents the mean for data points measured in triplicate. Values for *V*_max_ and *K_m_* were used to calculate the turnover number, *k*_cat_, and catalytic efficiency, *k*_cat_/*K_m_*, and are listed in the *table* on the *right. Error bars*, S.E. *C*, *cartoon representation* of the crystal structure of the USP15 catalytic core with catalytic triad residues shown as a *green stick representation* and active-site loops and key secondary structure elements *labeled. D*, *B*-factor “*putty representation*” of USP15-D1D2 highlighting the variation of *B*-factors where the *thickness* is proportional to its local *B*-factor and thus its flexibility and is *color-coded blue* to *red* (for lowest to highest *B*-factors). The approximate location of the distal ubiquitin is modeled as a *semitransparent surface representation* (*red*) into the S1 binding site and shows that the finger region will need to flex to accommodate ubiquitin.

USP11 typically engages in different protein interactions (23). USP15, USP4, and USP11 share the same overall domain structure, including an N-terminal domain present in USPs (DUSP) and ubiquitin-like (UBL) domain followed by a protease domain that harbors a ∼300-amino acid insertion predicted to contain a second UBL domain (Fig. 1*A*). These ancillary domains affect the catalytic function in different ways, whereby they regulate the catalytic activity of USP4 (24), whereas USP11's activity is not significantly modulated by their presence using a model substrate (17, 24). The role of ancillary domains in USP15 is less clear, with only a minor regulatory role on catalysis attributed to the USP15 DUSP–UBL domains so far (24). We and others previously determined crystal structures of the USP15 N-terminal domains (15, 16), but no structural information on the catalytic domain is available at present.

Here, we describe the structure of the USP15 catalytic core and compare it with the structure of paralog USP4 (24). Based on this analysis, we highlight key differences that may be responsible for altered specificity and regulation and propose a hypothetical model for conformational changes between “open inactive” and “closed catalytically competent” conformations. Furthermore, we determined the structure of a USP15–mitoxantrone complex, a Food and Drug Administration–approved antineoplastic drug (25), which has multiple cellular targets (26), including USP11 (25), that shows a novel binding mode for a USP ligand. Together, these findings allow novel insights into the USP15 structure, substrate recognition, and differences between close USP paralogs, and additionally they provide tools for structure-based drug design.

## Results

### The USP15 catalytic core structure displays a misaligned catalytic triad

To determine the structure of the USP15 catalytic domain, we designed a construct spanning the catalytic core region (USP15-D1D2; Fig. 1*A*), with the insert harboring a predicted UBL domain replaced by a short linker (based on the USP8 structure, PDB entry 2GFO (27)). Enzyme kinetic analysis of the catalytic core compared with full-length USP15 (FL-USP15) using the fluorogenic model substrate ubiquitin-7-amido-4-methylcoumarin (AMC) revealed that the kinetic parameters between the two are comparable, with similar *K_m_* and *k*_cat_ values (Fig. 1*B*). This showed that USP15-D1D2 fully retained its activity and suggests that the ancillary domains do not play a significant regulatory role in catalysis. USP15-D1D2 was then subjected to sparse matrix crystallization screening, and crystals were obtained in conditions using 0.1 m Tris-Cl, pH 8.5, 20% PEG 2000 (see “Experimental procedures”). After optimization, crystals of space group P2_1_ diffracted to 2 Å resolution and contained one molecule in the asymmetric unit. The structure was solved by molecular replacement using coordinates of paralog USP4 (PDB entry 2Y6E (24)) as a search model (data collection and refinement statistics are shown in Table 1). The USP15 catalytic core displays the characteristic USP protease domain fold, including a finger, palm, and thumb region (Fig. 1*C*). A C-terminal tail structure unique to USP15 is rich in aromatic residues and folds back onto the catalytic core in the structure and is located on the opposite face to the distal ubiquitin S1 binding pocket. The active-site loop regions between helices α5 and α6, also referred to as the switching loop (SL; including residues Pro^342^–Gln^353^; PQFSGYQQQDCQ), and residues between β12 and β13, referred to as blocking loop 1 (BL1; residues Ser^810^–Arg^816^; SYSRYMR) that engage in USP distal ubiquitin binding are partially flexible (unrestricted by crystal contacts and not visible in the electron density; indicated as *dotted lines* in Fig. 1*C*). As a consequence, the free enzyme structure of USP15 has a largely open catalytic channel conformation for accommodating the distal ubiquitin tail that harbors the two C-terminal glycines. Modeling of ubiquitin into the central binding cavity by superposition of the USP2–ubiquitin complex structure (PDB entry 2HD5 (28)) reveals that additional conformational changes in the finger region, including the zinc finger ribbon linking the two subdomains D1 and D2, are required to accommodate the distal ubiquitin core and avoid steric clashes (Fig. 1*D*). Mapping *B*-factors indicative of mobile regions onto the structure reveals that residues likely to undergo conformational changes upon substrate binding also display higher *B*-factors (Fig. 1*D*).

**Table 1:** **Crystallographic data collection and refinement statistics**

	USP15-D1D2 (USP15-free)	USP15-D1D2–mitoxantrone
**Data collection**		
Space group	P2_1_	P2_1_
Cell dimensions		
*a*, *b*, *c* (Å)	48.51, 62.62, 62.04	62.07, 94.39, 63.29,
β (degrees)	104.97	90.08
Resolution (Å)	1.98	2.09
*R*_merge_	0.111 (1.060)*^a^*	0.102 (1.619)
*R*_pim_	0.074 (0.711)	0.052 (0.87)
*I*/σ*I*	4.7 (1.7)	9.9 (1.57)
CC1/2	0.991 (0.527)	0.998 (0.668)
Completeness (%)	97.7 (86.3)	98.8 (98.5)
Redundancy	3.0 (3.0)	4.8 (4.9)
Wilson *B*-factor (Å^2^)	33.0	38.1

**Refinement**		
Resolution range (Å)	46.54–1.98	62.07–2.09
No. of reflections	24825	42810
*R*_work_/*R*_free_	0.198/0.234	0.203/0.251
No. of atoms	2734	5319
Protein	2602	5129
Other	1	37
Water	131	153
*B*-factors (Å^2^)		
Protein	48.1	59.0
Ligand		88.3
Water	50.5	50.5
Root mean square deviations		
Bond lengths (Å)	0.007	0.007
Bond angles (degrees)	0.794	0.904

*^a^* Values in parentheses are for the highest-resolution shell.

Interestingly, the USP15 structure shows the catalytic triad in an inactive conformation with the catalytic cysteine (Cys^269^) in the catalytic cleft loop between β1 and α1 (CCL; residues Ser^263^–Phe^270^; SNLGNTCF) located ∼10 Å away from the catalytic histidine (His^862^) (Fig. 1*C*). The CC loop also displays higher *B*-factors compared with surrounding residues, indicating that this region can readily adopt different conformations. Equally, residues in the blocking loop 2 between β15 and β16 (BL2; Gly^856^–Gly^860^; residues GGMGG) are partially flexible and associated with higher *B*-factors, suggestive of higher mobility. In contrast to Cys^269^, the side chains of catalytic triad residues His^862^ (on β16 at the C-terminal end of BL2) and Asp^879^ (end of β17) are “pre-aligned” for catalysis and within hydrogen-bonding distance (Fig. 1*C*).

### Differences between USP4 and USP15

**Figure 2.**
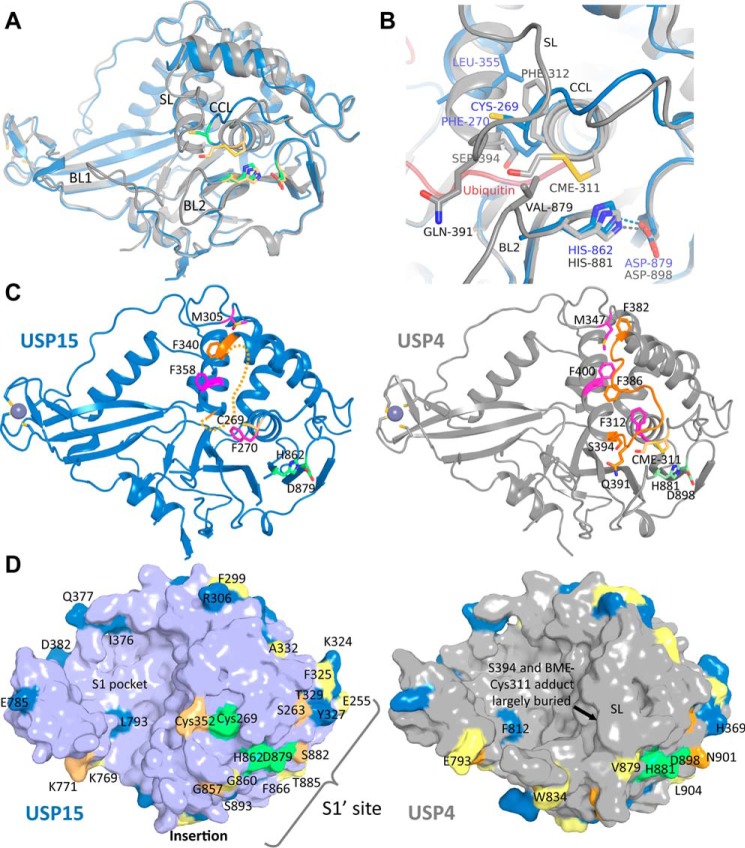
**Comparison of USP4 and USP15 catalytic core crystal structures.** *A*, superposition of *cartoon representations* of the USP15 structure (in *blue* with catalytic triad residues in *green*) and the USP4 structure with β-mercaptoethanol bound to the catalytic cysteine (in *gray* with catalytic triad residues shown as *sticks* in *wheat*; PDB ID 2Y6E (24)). Note the differences in the catalytic triad residues and surrounding loop regions CCL, SL, BL1, and BL2. *B*, *close-up view* of the active-site region showing the different conformations of USP15 (*blue*) and USP4 (*gray*) residues in the CCL, SL, and BL2. The approximate location of the ubiquitin C-terminal tail is depicted in a *red semitransparent cartoon representation. C*, conformational differences of selected *labeled residues* shown in *stick representation* in USP15 (in *blue* on the *left*) and USP4 (in *gray* on the *right*). Equivalent residues in both structures located in the switching loop SL are *highlighted* in *orange* (note that in USP15, the SL is largely flexible, indicated by a *dotted line*), whereas other intervening residues are *highlighted* in *magenta*. The catalytic cysteines are depicted in *wheat*, and the histidine and aspartate of the catalytic triad are *colored* in *green. D*, *surface representations* of USP15 (*left*) and USP4 (*right*) catalytic cores. Residues that are dissimilar between the two paralogs are *highlighted* in *yellow*, residues with weakly similar properties are *colored* in *orange*, and residues with similar properties are *colored* in *blue*. The *light blue* or *gray* background, respectively, denotes fully conserved residues between USP15 and USP4. Catalytic triad residues are *highlighted* in *green*, and selected residues are *labeled*. Note the differences in conservation between the large distal ubiquitin-binding cavity (S1 pocket) and the S1′ regions in both structures.

USP15 shares 56.9% sequence identity with paralog USP4 over the entire length of its sequence, 57.8% sequence identity in the catalytic domain, and 77.4% in the catalytic core region D1D2. We therefore compared our structure with the available structure of the USP4 catalytic core (USP4-D1D2; PDB entry 2Y6E (24)) by superimposing the two structures (root mean square deviation of 0.916 Å over 315 equivalent Cα positions). In contrast to USP15, the catalytic triad in the USP4 structure is pre-aligned in a catalytically competent configuration. Both structures were determined in the absence of ubiquitin; USP4 was captured with a β-mercaptoethanol (BME) molecule covalently linked to the catalytic cysteine (Fig. 2, *A* and *B*). These differences in the catalytic competency states of the catalytic triad coincide with different conformations of USP15 Phe^270^ compared with USP4 Phe^312^, the neighboring residue to the catalytic cysteine in the CC loop (Fig. 2, *B* and *C*). In our USP15 structure, the switching loop is partially flexible (Fig. 1*C*), whereas in the USP4 structure it is ordered, possibly aided by interactions with neighboring molecules in the crystal lattice (24). In addition, the C-terminal end of the switching loop and start of helix α6 are different in the two structures: in USP15 following residues of the SL, helix α6 starts with residue Leu^355^, whereas in USP4, equivalent residues before this leucine adopt an additional helical turn starting with Ser^394^ (Fig. 2*B*). In USP15, Gln^349^ in the SL is flexible, but in USP4, the equivalent Gln^391^ reaches across the channel possibly aided by interactions with the BME molecule involving main chain NH and carbonyl groups from Gln^391^ and Gln^392^ and Val^879^ in the BL2. As a result, the ubiquitin C-terminal GG tail–binding channel is mostly open in USP15, whereas it is closed in the USP4 structure (Fig. 2, *C* and *D*).

At the N-terminal end of the SL, the side chain of Phe^340^ (USP15) adopts a conformation (“in”), which is different from Phe^382^ in USP4 (“out”), whereas USP15 Pro^342^ and Gln^343^ adopt conformations similar to those of their USP4 counterparts. In turn, USP15 Met^305^, which packs against the side chain of Phe^340^, also adopts different conformations compared with USP4 (Met^347^ and Phe^382^) (Fig. 2*C*). These differences also coincide with different side chain conformations of USP15 Phe^358^ (out) and USP4 Phe^400^ (in) in helix α6, respectively (Fig. 2*C*). The preceding α5 helix is located further “in” in USP15, whereas in USP4, the ordered SL contains a helical turn (USP4 residues 384–387 PQFS), whereby the USP4 Phe^386^ side chain packs against residues Leu^397^, Phe^312^, and Phe^400^ in the core. The corresponding residues of the same amino acid sequence in the USP15 structure (Pro^342^–Ser^345^) are partly defined in the electron density due to flexibility. A USP15–USP4 amino acid sequence alignment in the SL region reveals the only difference to be a cysteine residue (USP15 Cys^352^), which replaces a serine (Ser^394^) in USP4 (Fig. 2*D* and Fig. S1). USP15 SL residue Cys^352^ is conserved across an alignment of USP15 amino acid sequences, but in the crystal structure, it is not well-defined and therefore was not modeled and assumed to be flexible.

We then mapped all residues that differ between USP15 and USP4 across the catalytic core onto the USP15 surface and vice versa, which revealed that residues in the distal ubiquitin-binding pocket are highly conserved between USP15 and USP4 (Fig. 2*D*). Greater variability in amino acids is observed in the S1′ binding region, which engages a proximal ubiquitin moiety in a polyubiquitin substrate or a different target substrate conjugated to the C-terminal tail of ubiquitin (Fig. 2*D*). Of note is BL2, which in USP15 (GGMGG) is highly mobile due to the presence of four glycines, whereas BL2 in USP4 contains fewer glycine residues (GAMGV) and adopts a different conformation (Fig. 2*A* and Fig. S1), although both display high *B*-factors, highlighting their mobility. Substitution of glycine for Val^879^ in USP4 may contribute to different BL2 conformations, as Val^879^ is positioned at the rim of the ubiquitin GG tail–binding channel (Fig. 2*B*). Other differences in the S1′ area cluster in the loop region between α4 and α5 (USP15 324–329 (KFSYVT) *versus* USP4 366–371 (RDAHVA)), which is close to the linker region that connects the catalytic core to the N-terminal UBL domain. There, USP15 Phe^325^, Ser^326^, and Tyr^327^ are replaced by USP4 Asp^367^, Ala^368^, and His^369^, respectively. Other changes in this area include USP15 Ser^263^ (USP4 Gly^305^), USP15 Ser^882^ (USP4 Asn^901^) and USP15 Thr^885^ (USP4 Leu^904^) (Fig. 2*D*). In addition to the S1′ region, other significant structural differences occur on the surface of helix α7 at the opposite site of the distal ubiquitin-binding pocket with a charge reversal (USP15 Glu^391^ *versus* USP4 Lys^433^) and differences in the location of hydrophobic and hydrophilic residues (USP15 Leu^398^-Lys^399^ *versus* USP4 Arg^440^-Leu^441^).

**Figure 3.**
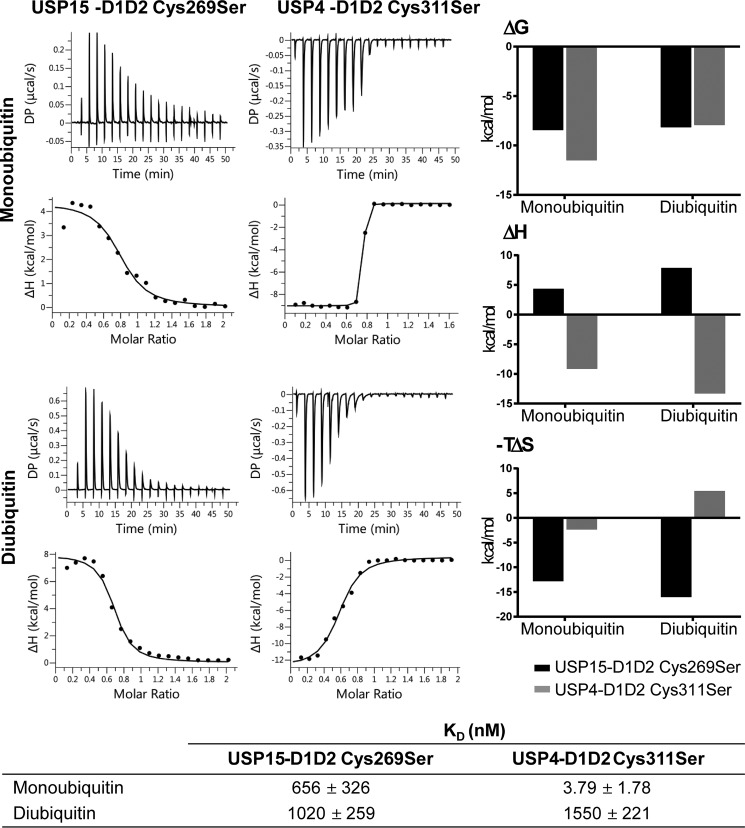
**Monoubiquitin and linear diubiquitin binding to USP4 and USP15 active-site mutants.** ITC analyses of raw data measured at 25 °C and binding isotherms fitted to a one-site binding model of USP15-D1D2 C269S and USP4-D1D2 C311S with monoubiquitin and linear diubiquitin, respectively. Respective dissociation constants are listed in the *table below*, and associated Δ*G*, −*T*Δ*S*, and Δ*H* values are graphically depicted on the *right* to highlight different contributions to the binding.

**Figure 4.**
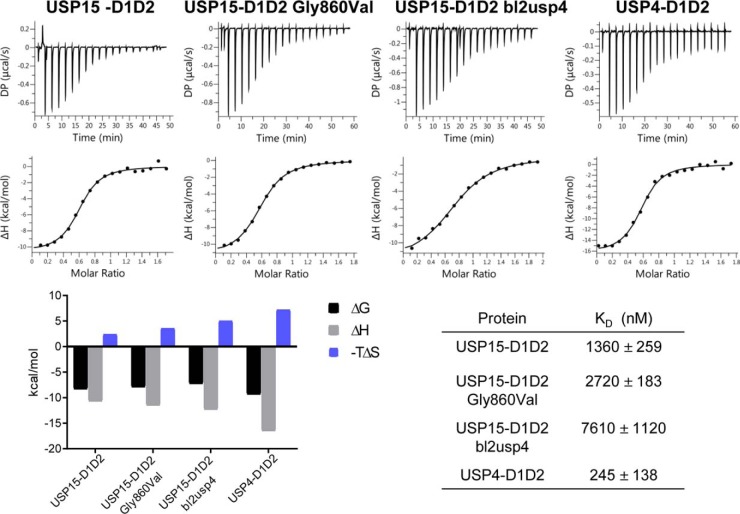
**Influence of blocking loop 2 mutations on the ubiquitin-binding behavior of USP15.** ITC raw data and binding isotherms fitted to a one-site binding model of USP15-D1D2, USP15-D1D2 G860V, USP15-D1D2 bl2usp4, and USP4-D1D2 with monoubiquitin at 37 °C. The respective dissociation constants are given in the *table below* on the *right*, and the associated thermodynamic parameters Δ*G*, Δ*H*, and −*T*Δ*S* are graphically represented on the *left*.

To evaluate the substrate- and product-binding behavior of the USP15 and USP4 catalytic cores, we measured dissociation constants of inactive mutants USP15-D1D2 C269S and USP4-D1D2 C311S with monoubiquitin and linear diubiquitin (occupying either the S1 or both S1 and S1′ pockets, respectively). Remarkably, the results showed that monoubiquitin binds significantly tighter to USP4, whereas for linear diubiquitin, the dissociation constant for the interaction with USP15 was of the same order of magnitude compared with USP4 (Fig. 3). Interestingly, the enthalpy and entropy contributions associated with the binding events differed significantly, with USP15 displaying endothermic binding behavior, whereas USP4 displayed exothermic binding behavior for mono- and linear diubiquitin at 25 °C. We then further investigated the molecular basis of these differences through mutational analysis by swapping residues in the USP15 BL2 for the respective USP4 residues. These ITC experiments were carried out at 37 °C to record good signal/noise ratios for the USP15-D1D2 G860V and USP15-D1D2 bl2usp4 (G857A/G860V) mutants, which produced small heat change upon ubiquitin binding at 25 °C (data not shown). The USP15-D1D2 interaction with ubiquitin was exothermic under these conditions. These experiments showed that thermodynamic parameters Δ*H* and Δ*S* for the interaction of monoubiquitin with the USP15-D1D2 G860V and USP15-D1D2 bl2usp4 (G857A/G860V) mutants gradually changed with the stepwise substitution of the glycines in the BL2 approaching those obtained for USP4-D1D2 (Fig. 4). The difference in the dissociation constants for the interactions between active USP15-D1D2 and USP4-D1D2 and monoubiquitin was less pronounced in these experiments compared with the interaction with the catalytic Cys-to-Ser mutants. The SL has only one difference in the amino acid sequence between USP15 and USP4, and the SL mutant (USP15-D1D2 C352S) displayed similar binding parameters compared with USP15-D1D2 (Fig. S2).

### Comparison with other USPs

The majority of USP catalytic domain structures available to date were determined in complex with either ubiquitin or a covalent inhibitor including USP2 (28), USP4 (24), USP5 (29), USP7 (30), USP12 (31), USP18 (32), USP14 (33), USP21 (34), USP30 (35), USP46 (36), and CYLD (37). Structures that do not have a ligand or ubiquitin covalently bound to the catalytic cysteine are available for USP7 (PDB codes 1NB8, 2F1Z, 4M5X (30, 38, 39)), USP14 (PDB code 2AYN (33)), CYLD (PDB code 2VHF (40)), USP8 (PDB code 2GFO (27)), USP18 (PDB code 5CHT (32)), and USP12 (PDB codes 5K1B and 5K16 (41)). Among the latter structures, the USP15 catalytic core is most closely related to USP8 (46% sequence identity) followed by USP12 (26.32% sequence identity), whereas it is more distantly related to USP7 (21.99% sequence identity), USP14 (19.94% sequence identity), and CYLD (11.8% sequence identity).

**Figure 5.**
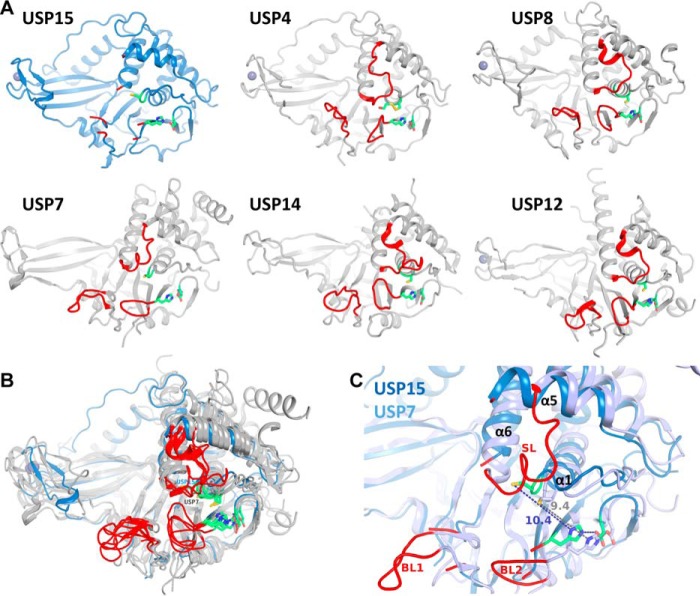
**Comparison with other USP catalytic core structures determined in the absence of ubiquitin.** *A*, crystal structures of USP15 (*blue*) and USP4 (PDB code 2Y6E (24)), USP8 (PDB code 2GFO (27), USP7 (PDB code 4M5X (38)), USP14 (PDB code 2AYN (33)), and USP12 (PDB code 5K16 (41)) shown in a *gray cartoon representation* with catalytic triad residues *highlighted* in *green* and BL1, SL, and BL2 loop regions each depicted in *red. B*, superposition of the same structures as seen in *A*, highlighting the most variable regions in the structures. *C*, superposition of the active-site region of USP15 (*blue*) and USP7 (*light blue*), the only known USP catalytic core structures whose catalytic triad is misaligned in a similar way in the free enzyme. Note the difference in switching loop conformations and the significant distances (Å) between the catalytic cysteines and histidines.

Apart from USP7, the catalytic triad residues in these structures are largely pre-aligned in close proximity, and the catalytic cysteine is located in the first turn of helix α1 (Fig. 5, *A* and *B*). In our USP15 structure, the distance between the catalytic cysteine thiol group (Cys^269^) and the catalytic histidine imidazole (His^862^) is about 10 Å, which is even slightly farther away than the catalytic cysteine in the USP7 free enzyme structures (Fig. 5*C*) and is part of an extended CC loop. In USP7, the conformational changes between free and ubiquitin-bound forms involve different conformations of the aromatic residue C-terminal to the catalytic cysteine in the CC loop (Phe^270^ in USP15 and Phe^312^ in USP4) (30, 42). USP7 Tyr^224^ flips in and out with concomitant conformational changes in the SL and CC loop, which together determine whether the position of the catalytic cysteine is pre-aligned in the catalytic triad. However, in USP7, the SL region has a different sequence and structure (Fig. 5*A* and Fig. S1) and is ordered in the free as well as ubiquitin suicide inhibitor–bound forms possibly aided by crystal contacts, whereas in the USP15 structure, the SL is flexible and largely not visible in the electron density. In a CYLD structure (PDB code 2VHF), the SL is also observed as flexible in the crystal structure, but otherwise, the SL is ordered in other USP structures. The only highly conserved residue in the SL region among these USPs is the glutamine residue (part of the so-called USP QQD box (43)) that closes over the ubiquitin GG tail channel in ubiquitin-bound USP structures, including USP2 (28), USP7 (30), USP21 (34), USP46 (36), USP12 (31), and USP14 (33); in USP30, it is a glutamic acid, Glu^159^ (35).

### Mitoxantrone inhibits the activity of USP15 and occupies the S1′ region of the catalytic core

**Figure 6.**
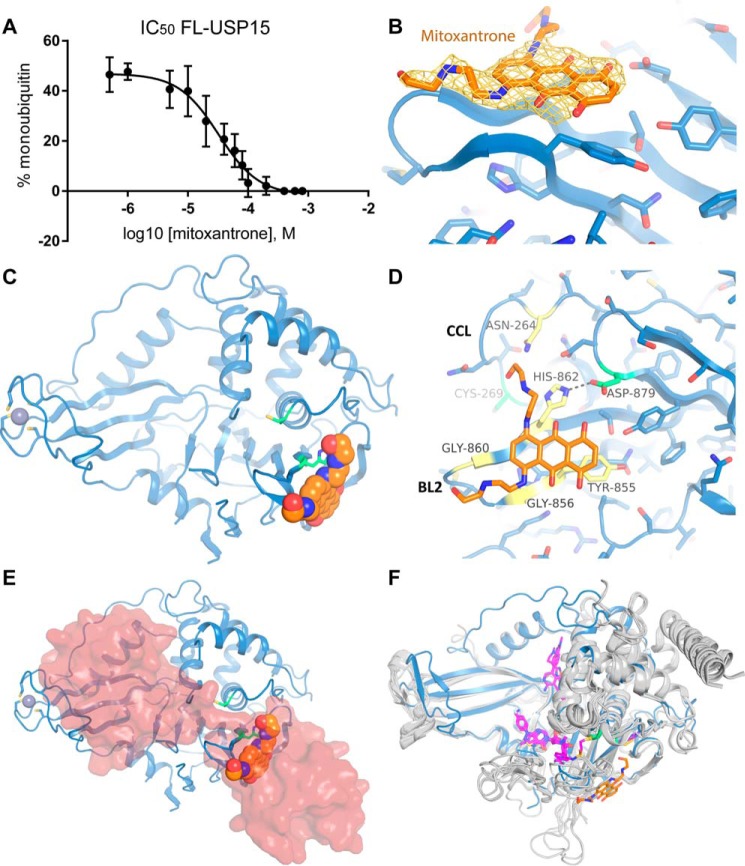
**Inhibition of USP15 by mitoxantrone.** *A*, IC_50_ curve for mitoxantrone as an inhibitor of USP15 using diubiquitin gel shift cleavage assays. *Error bars*, S.E. *B*, *mF*_obs_ − *DF*_calc_ omit electron density map calculated with the mitoxantrone molecule removed contoured at 2.0σ with the density shown in *light orange* and corresponding final model in an *orange stick representation. C*, *cartoon representation* of the USP15–mitoxantrone complex crystal structure. The USP15 protease domain is depicted in *blue* with catalytic triad residues in *green* and mitoxantrone shown in a *space-fill representation* in *orange. D*, *close-up view* of the molecular basis of the interaction. Key residues involved in the interaction are *labeled* and shown as *yellow sticks*. Otherwise, the *color code* is the same as in *C. E*, same representation of the USP15–mitoxantrone complex as in *C* with a diubiquitin substrate shown as a *semitransparent surface* modeled into the active site based on the orientation seen in the crystal structure of the USP30–Lys^6^-diubiquitin complex (PDB code 5OHP (35)) to highlight clashes with mitoxantrone binding in the S1′ pocket. *F*, superposition of available crystal structures of USP7 (*gray*) in complex with small-molecule inhibitors in *magenta stick representations* (PDB codes 5N9R, 5N9T, 5NGE, 5NGF, 5UQV, 5UQX, and 5WHC (49–51)) and USP2 (*gray*; PDB code 5XU8 (52)), highlighting the interaction locations in predominantly the S1 pocket and active-site channel. USP15 is depicted in a *blue cartoon representation* with mitoxantrone in *orange*. Catalytic triad residues are depicted in *green stick* representations.

We subsequently tested whether USP15 is inhibited by mitoxantrone using a diubiquitin gel shift cleavage assay, as this agent has previously been shown to inhibit the homolog USP11 (25). We determined that USP15 is weakly inhibited by mitoxantrone with an IC_50_ value of 33 ± 11 μm (Fig. 6*A* and Fig. S3). Co-crystallization trials in the presence of excess mitoxantrone yielded crystals of intense blue color that diffracted to 2.1 Å resolution (data collection and refinement statistics are shown in Table 1). After molecular replacement, an additional planar shape of electron density was visible in the vicinity of the BL2 region near the catalytic histidine that corresponded to the anthracenedione structure of mitoxantrone (Fig. 6*B*). The catalytic triad in this different crystal form is also misaligned in both USP15 copies present in the asymmetric unit in a similar configuration to the USP15-free enzyme structure (Fig. 6*C*). This USP15–mitoxantrone complex structure revealed predominantly hydrophobic interactions between mitoxantrone and USP15 residues Tyr^855^, Gly^856^, Gly^860^, His^862^, which are located in the BL2 region (Fig. 6*D*). In addition, one of mitoxantrone's side arms contacts the CC loop Asn^264^, although the density is weaker compared with the mitoxantrone anthracenedione core (Fig. 6*B*). There are no interactions of mitoxantrone with other neighboring USP15 molecules in the crystal lattice. This binding site coincides with the S1′ region of USP15, suggesting a rationale for the inhibition observed (Fig. 6*E*). In this position, mitoxantrone is expected to compete with substrate interactions, as indicated by modeling a diubiquitin molecule into the binding site based on a superposition with a USP30 C77A Lys^6^-linked diubiquitin structure (35), the closest available USP structure in complex with a substrate (Fig. 6*E*). Superposition of the USP15-free and mitoxantrone-bound structures revealed that the BL2 becomes ordered, but no other major conformational changes are observed upon mitoxantrone binding.

## Discussion

There are 58 USPs in the human genome, but only for a small subset is structural information on their catalytic domains available to date. Most structures have been determined in complex with ubiquitin or ubiquitin-based inhibitors. Here, we determined the structure of the USP15 catalytic core domain, which reveals a structure with a misaligned catalytic triad, with the catalytic Cys^269^ and His^862^ separated by a large distance and active-site loop conformations not previously observed for any other USP. Our USP15 structure compared with USP4 (24) revealed several differences in the active-site and substrate-binding regions, but the comparison may also suggest an activation pathway upon ubiquitin binding as outlined below.

The USP15 structure reported here displays a catalytically incompetent open conformation of the ubiquitin C-terminal tail–binding channel with active-site loops not engaged in crystal contacts, whereas a “closed” form of USP4 was previously captured with a pre-aligned catalytic triad (24). The structural comparison, partial functional overlap, high sequence conservation in the S1 pocket, and the assigned roles and conformations of certain residues around the active site in other USPs suggest that the two structures largely represent open (inactive) and closed (active) conformational states, as the bound BME in USP4 co-localizes with the expected position of the ubiquitin tail (Fig. S4), and crystal packing interactions may have promoted order in the USP4 switching loop. Substrate binding is likely to result in active-site rearrangement and alignment of the catalytic residues, which at least in part may coincide with conformational changes observed between the two structures.

In this scenario, possible conformational changes in USP15 upon substrate binding leading to the alignment of the catalytic triad can be summarized as follows: (i) flexible-to-order transition of BL1 and (ii) flexible-to-order transition in parts of the C-terminal end of the switching loop, including Glu^354^ repositioning to form a salt bridge with ubiquitin Arg^72^. The flexible USP15 Cys^352^ would be expected to adopt an α-helical conformation extending α6, as in USP4 the equivalent residue Ser^394^ is located on an additional α6 helical turn and faces inward, forming a hydrogen bond with Ser^871^ (USP15 Ser^852^). In this conformation, USP15 Cys^352^ would displace the Phe^270^ side chain in the CC loop to adopt a similar conformation as USP4 Phe^312^. This in turn would require displacement of the catalytic Cys^269^ to avoid steric clashes and would result in the alignment of the catalytic triad. As is typical for USP structures, the USP15 SL Gln^349^ is expected to reposition to close over the ubiquitin GG tail–binding channel. (iii) At the N-terminal end of the SL, a flexible-to-order transition of USP15 Phe^344^ (USP4 Phe^386^) could result in repacking of other hydrophobic residues, predominantly USP15 Phe^358^, Phe^340^, and Met^305^, and pushing helix α5 outward. The hydrophobic residues involved are highly conserved in USP15 and USP4 across species, and a USP4 SL F386G mutant was shown to increase the catalytic activity of the USP4 catalytic domain (USP4-CD) activity by affecting ubiquitin on and off rates (24).

**Figure 7.**
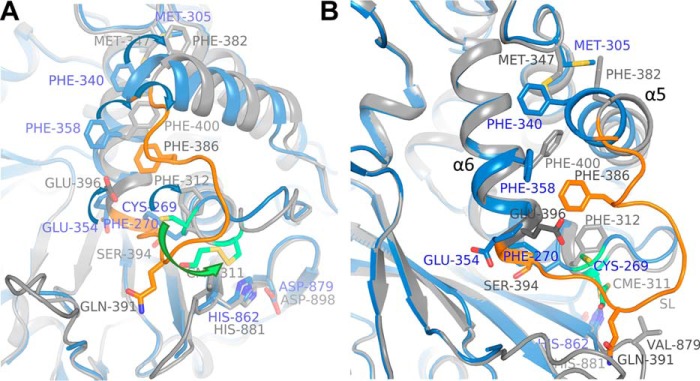
**Hypothetical model of an activation mechanism to align the USP15 catalytic triad upon substrate binding.** *A*, superposition of USP15 (*blue*) and USP4 (*gray*; PDB code 2Y6E (24)) crystal structures with possible conformational changes upon substrate binding to align the remote Cys^269^ into an active conformation indicated based on the two structures. Hypothetical conformational changes of selected residues in the SL (*orange* in both structures), helices α5 and α6, CC loop, and surrounding regions are indicated by *arrows* between equivalent residues in the sequences. The active-site cysteines are both depicted in *green stick representations* (BME adduct in USP4). *B*, different view of *A* to highlight conformational differences between the two structures. The *color code* is as in *A*.

A hypothetical model of this proposed activation mechanism upon ubiquitin binding for USP15 is shown in Fig. 7. In addition, flexing of the finger *versus* palm regions will need to occur upon substrate binding to avoid steric clashes. Further investigations, such as the determination of a USP15 substrate- or product-bound structure and molecular dynamics simulations will be required to confirm this mechanism.

Interestingly, differences between free and ubiquitin-bound forms of USP7 also rely on conformational changes in the switching and CC loops involving aromatic side chains, but the involved residues and adopted conformations of the loop regions, especially the switching loop, are different compared with the proposed changes between USP15-free and substrate-bound forms (42). Examples of unproductive misaligned catalytic triads are also found among other deubiquitinating enzyme families, such as ubiquitin C-terminal hydrolases (UCH family) that involve conformational changes of aromatic residues and OTULIN (OTU family), but in these cases, these are mainly restricted to realignment of the catalytic histidines that act as a general base (44).

On the other hand, we noted clear differences between USP15 and USP4 that could contribute to different modes of regulation and substrate specificities in line with previous reports (24). The product (monoubiquitin)- and model substrate (diubiquitin)-binding behavior determined by ITC indicated that product inhibition plays a larger role for USP4 than USP15 consistent with previous data, whereas both paralogs engage a linear diubiquitin substrate with similar equilibrium affinities. The dissociation constant for monoubiquitin binding to the USP4-D1D2 catalytic core is comparable with the one reported previously for USP4-CD and FL-USP4 (24). For USP15-D1D2, both binding events are associated with endothermic binding enthalpies at 25 °C, indicating an entropically driven process. In contrast, USP4 mono- and linear diubiquitin interactions are enthalpically driven. In the absence of USP4 and USP15 ubiquitin-bound structures, the molecular bases for these differences are difficult to rationalize at present, as multiple factors will contribute, such as the release of bound water molecules, conformational changes associated with the binding events, and differences in interacting residues.

However, we identified that the BL2 influences the binding parameters. The USP15 BL2 is more flexible due to the presence of an additional two glycine residues compared with USP4. Within the USP4 BL2, Val^879^ packs against Glu^391^ and is close to the expected position of the ubiquitin tail. When we substituted the corresponding Gly^860^ in USP15 with a valine, the USP15-D1D2 G860V mutant ubiquitin-binding parameters were altered, confirming that this residue is involved in ubiquitin binding. A USP15-D1D2 G857A/G860V mutant (USP15-D1D2 bl2usp4) displayed a further change in the Δ*H* and Δ*S* parameters associated with ubiquitin binding toward those obtained for USP4 with a more favorable enthalpic contribution, but less favorable entropic contribution, which may reflect additional order in the BL2 through interactions with the ubiquitin tail (Fig. 4). The BL2 glycines in USP15 may therefore contribute to differences in the ubiquitin discharge behavior between USP15 and USP4 (24). The difference in sequence in the switching loop (USP15 Cys^352^ to USP4 Ser^394^) had little impact on the ubiquitin-binding properties (Fig. S2). It is evident that additional residues at the interface, and/or noninteracting regions through long-range effects, contribute to USP4 and USP15's ubiquitin-binding behavior.

In USP4, the switching loop is ordered, possibly aided by crystal contacts, and interacts with the N-terminal DUSP domain (24), whereas in USP15, the SL is largely flexible in the free enzyme structure, and the DUSP domain is not known to interact with the catalytic core domain, probably due to differences in the N-terminal regions (15, 16, 24). In contrast to USP4, we show that kinetic parameters of USP15-D1D2 are very similar to FL-USP15; hence, the catalytic core does not rely on additional regions for its catalytic turnover, which is largely consistent with data collected previously on USP15-CD and FL-USP15 (24) that showed only a minor effect upon removal of the N-terminal domains. USP15's sigmoidal kinetic behavior is consistent with the occurrence of conformational changes (45). The USP4 catalytic domain also displays sigmoidal kinetic behavior (24).

The only other USP shown to have a misaligned catalytic triad in a structure to date that requires significant conformational rearrangements for activation is the catalytic domain of USP7 (USP7-CD) (30, 38). However, in contrast to USP15, the USP7-CD only displays limited activity, and the C-terminal UBLs and C terminus are required for full catalytic competency, whereby they interact with the SL to promote conversion of the catalytic loop to an α-helical conformation and catalytic triad alignment (46–48). The activity of USP15, on the other hand, is only minimally influenced by the presence of additional domains, as observed by us and others (24), but also has a misaligned catalytic triad, from which we infer a different mechanism of regulation. It is possible that, like USP15 and USP7, other USPs may also have flexible CC loops that can adopt catalytically competent and incompetent conformations, as few free enzyme structures of USPs have been determined to date, and crystal structures may preferentially select one conformation. Moreover, binding partners may also influence the competency state of the catalytic triad.

Furthermore, we show that mitoxantrone weakly inhibits USP15 and determined a mitoxantrone–USP15 complex structure. Only recently, the first USP–inhibitor complex structures have been published, namely for USP7 (49–51) and USP2 (52). These either occupy the ubiquitin GG tail–binding cleft (noncovalently or covalently linked to the active-site cysteine) (51), compete with distal ubiquitin binding (50), or act noncompetitively (52). Here, we describe a novel binding mode for a USP whereby a small molecule interacts with the S1′ region and therefore may interfere with proximal substrate moiety interactions (Fig. 6*F*). Mitoxantrone is a promiscuous binder with several targets described to date, including DNA-bound topoisomerase II β (53), focal adhesion kinase (FAK) (54), human serine/threonine kinase Pim1 (26), and USP11 (25). In available crystal structures of PIM1 (PDB codes 4RC2, 4RBL, and 4I41 (26)) and type II topoisomerase (PDB code 4G0V (55)), mitoxantrone binding modes also predominantly engage the anthracenedione ring structure mediating interactions with hydrophobic residues.

Together, our data shed new light onto the conformational diversity of ubiquitin-free USP structures, differences between close paralogs, regulation through incompetent conformational states, and modes of small-molecule interactions, which enhances our molecular understanding of their modes of action and will aid structure-based molecular probe and drug design efforts.

## Experimental procedures

### Cloning, mutagenesis, expression, and protein purifications

Based on bioinformatical analysis, a human USP15 catalytic core domain construct, USP15-D1D2 (residues 255–919 Δ440-756) was designed and cloned into pET21d via NcoI and NotI restriction sites. A small linker (ASTSK) corresponding to the USP8 sequence was used to link the two catalytic subdomain halves, D1 and D2, and replace the insertion of ∼315 residues that is predicted to contain a high percentage of disorder. FL-USP15 (UniProtKB identifier Q9Y4E8-2) was cloned via SacI and HindIII restriction sites into the pCold-I expression vector. USP15 mutants C269S, C352S, and G860V were created using primers GTAACTTGGGAAATACGTCTTTCATGAACTCAGCTATTCAG/CTGAATAGCTGAGTTCATGAAAGACGTATTTCCCAAGTTAC, AAGCTAACAGTTCTTGACTGTCTTGCTGCTGATATCC/GGATATCAGCAGCAAGACAGTCAAGAACTGTTAGCTT, and CTATGGAGGGATGGGAGTAGGACACTATACTGCTTTTG/CAAAAGCAGTATAGTGTCCTACTCCCATCCCTCCATAG, respectively, following the QuikChange mutagenesis protocol. The USP15-D1D2 bl2usp4 loop swap mutant (G857A/G860V) was created by swapping USP15(Gly^857^–Gly^860^; GMGG) for the corresponding USP4 sequence AMGV. The USP4-D1D2 catalytic core (residues 294–963 Δ484–775) with the insertion removed and replaced with the USP8 ASTSK sequence analogously to USP15 was cloned into the pProEx-HTb expression vector using BamHI and HindIII restriction sites. The USP4 C311S mutant was created using primers GGAAACCTGGGAAACACCAGCTTCATGAACTCCGCT/AGCGGAGTTCATGAAGCTGGTGTTTCCCAGGTTTCC.

All USP constructs were expressed in 2YT broth medium using the *Escherichia coli* BL21-CodonPlus strain. Cells were grown at 37 °C to *A*_600_ of ∼0.6 and further grown after induction by 0.5 mm isopropyl 1-thio-β-d-galactopyranoside overnight at 25 °C (USP15-D1D1 and USP4-D1D2 constructs) or 48 h at 10 °C (FL-USP15) after induction. Cells were harvested, lysed by sonication, and clarified by centrifugation. USP proteins were lysed into 50 mm Tris-Cl, pH 7.5, 300 mm NaCl, 5 mm imidazole, and 1% glycerol (10% for FL-USP15). Proteins were then loaded onto a HiTrap chelating column precharged with nickel ions and eluted by an imidazole gradient. This was followed by size-exclusion chromatography using a buffer of 20 mm Tris-Cl, pH 7.5, 150 mm NaCl, and 1% glycerol on a Superdex200 16/60 column (GE Healthcare). Fractions were analyzed by SDS-PAGE, and relevant fractions were combined and concentrated for further use.

### Enzymatic and inhibition assays

Kinetic parameters for FL-USP15 and USP15-D1D2 were derived from deubiquitinating assays with concentrations in the range of 0.1–1.5 μm ubiquitin-AMC as the fluorogenic substrate and 38 nm FL-USP15 or USP15-D1D2 in 150 mm NaCl, 50 mm Tris-Cl, pH 7.5, 1 mm DTT. Measurements were taken in 30-μl final volumes in triplicate in 384-well black plates (Nunc) read with an EnVision 2104 multilabel plate reader at 25 °C using an excitation wavelength of 355 nm and an emission wavelength of 426/428 nm. Measurements were taken every minute for the first 20 min and then every 2 min for the next 30 min and subsequently at increasing intervals of 5, 10, and 20 min. Curves measured in triplicate were fitted using nonlinear regression analysis in GraphPad prism software (allosteric sigmoidal model using the equation, *Y* = *V*_max_ × X*^h^*/(*K*_half_*^h^* + *X^h^*)) to establish *K*_half_ (referred to as *K_m_* throughout for consistency with the literature) and *k*_cat_ values.

Gel shift linear diubiquitin cleavage assays were carried out in triplicate in two independent experiments at 25 °C in 50 mm Tris-Cl, pH 7.5, 300 mm NaCl, 1% glycerol, and 1 mm DTT. Mitoxantrone was solubilized in the assay buffer, and its concentration was determined by measuring the absorbance at 682 nm using an extinction coefficient of 8360 m^−1^ cm^−1^. Reactions were initiated by the addition of linear diubiquitin to FL-USP15, resulting in final concentrations of 5 μm linear diubiquitin and 400 nm USP15, respectively. The reaction was stopped by the addition of SDS-PAGE loading buffer. Linearity tests were completed to establish the linear range of the reaction. For the IC_50_ curve, mitoxantrone was added to FL-USP15 at specified concentrations in the range of 0.5–800 μm and pre-incubated for 30 min before the addition of linear diubiquitin. Reactions were stopped after 30 min and analyzed on 18% SDS-polyacrylamide gels. After staining, gels were scanned and then analyzed with the ImageJ software where relative amounts of diubiquitin and monoubiquitin for each point were determined. The mean monoubiquitin percentage was calculated and plotted using GraphPad Prism software using nonlinear regression analysis.

### Isothermal titration calorimetry (ITC)

ITC data were measured on a PEAQ ITC instrument (Malvern). Monoubiquitin or linear diubiquitin samples (200–600 μm) were titrated into the sample cell containing 20–60 μm USP15-D1D2, USP15-D1D2 C269S, USP15-D1D2 C352S, USP15-D1D2 G860V, USP15-D1D2 bl2usp4, USP4-D1D2, or USP4-D1D2 C311S samples in 150 mm NaCl, 50 mm Tris-Cl, pH 7.5, and 1% glycerol at a temperature of 25 or 37 °C. The spacing was typically 180 s, and a stirring speed of 750 rpm was used. Data were analyzed using the PEAK ITC analysis software (Malvern) fitting to a one-site binding model.

### Protein crystallization, data collection, and structure determination

Crystallization of USP15-D1D2 was carried out using a protein concentration of 4 mg/ml in gel filtration buffer at 20 °C in the presence and absence of inhibitors. Crystals using 0.1 m Tris-Cl, pH 8.5, and 20% PEG 2000 as crystallization mother liquor were obtained in the presence of mitoxantrone (1,4-dihydroxy-5,8-bis[2-(2-hydroxyethylamino)ethylamino]anthracene-9,10-dione) in gel filtration buffer at a ∼1:10 molar ratio and cryoprotected by soaking in a mother liquor supplemented with 10% glycerol, 5% ethylene glycol, 1.75% dioxane, and 1.5 mm pan-USP inhibitor PR-619. Crystals belonged to monoclinic space group P2_1_ with unit cell parameters *a* = 48.51 Å, *b* = 62.62 Å, *c* = 62.04 Å, and β = 104.97° and contained one molecule in the asymmetric unit. Data of these crystals were collected at the ESRF beamline ID30A-1 at a wavelength of 0.966 Å and 100 K. Data were processed using XDS (56) and AIMLESS (57), and the structure was solved by molecular replacement using coordinates from the human USP4 structure (PDB code 2Y6E (24)) as a search model with PHASER (58). The inhibitors were not observed in the electron density in these crystals, and we refer to this as the free enzyme structure (USP15-free).

USP15 crystals were also obtained by incubating the protein with a ∼1:15 molar excess of mitoxantrone before crystallization in the condition 0.1 m Na-Hepes, pH 7.0, 14% MPD at 20 °C. These crystals were flash-cooled after soaking in a cryoprotectant solution of 35% MPD supplemented with 1 mm mitoxantrone for data collection. Crystals belonged to space group P2_1_ with unit cell parameters of *a* = 62.07 Å, *b* = 94.39 Å, *c* = 63.29 Å, and β = 90.08° containing two molecules in the asymmetric unit and diffracted to 2.1 Å resolution. A data set (USP15–mitoxantrone) was collected at beamline I04 at Diamond Light Source at a wavelength of 0.97951 Å and a temperature of 100 K. Data collection statistics for both data sets are summarized in Table 1.

### Model building, refinement, and validation

Model building and adjustments were conducted using COOT (59). Structure refinement was carried out in PHENIX (60), and the quality of the model was assessed by MOLPROBITY (61). For the USP15 structure from the crystals with one molecule in the asymmetric unit (USP15-free), the final model consisted of 325 residues with residues in the BL1, SL, and BL2 loop regions and the C terminus not modeled due to flexibility. The USP15–mitoxantrone complex data set consisted of two molecules in the asymmetric unit, whereby one of the molecules clearly displays density for mitoxantrone in its vicinity (Fig. 6*B*). The final USP15–mitoxantrone complex structure consists of 331 and 309 residues in the two copies, respectively, with residues in the BL1 and SL regions and the C terminus not modeled due to flexibility. Further electron density indicated stacking interactions of additional mitoxantrone molecules against the USP15-interacting mitoxantrone molecule, but these were not modeled, as their orientation was unclear. In the final models, there are no Ramachandran outliers with 98% of residues (USP15-free) and 96% of residues (USP15–mitoxantrone) located in favored regions, respectively. Refinement statistics are summarized in Table 1. Figures were prepared in PyMOL (Schrödinger, LLC).

## Author contributions

S. J. W., H. E. G., J. E., and I. D. formal analysis; S. J. W., S. K. M., and S. G. C. validation; S. J. W., H. E. G., P. I., C. M., R. M., and S. K. M. investigation; S. J. W. methodology; S. J. W., J. E., and I. D. writing-original draft; I. D. conceptualization; I. D. data curation; I. D. supervision; I. D. funding acquisition; I. D. project administration.

## Supplementary Material

Supporting Information

## Footnotes

5 The abbreviations used are:

USP: ubiquitin-specific protease
ITC: isothermal titration calorimetry
BME: β-mercaptoethanol
UBL: ubiquitin-like
DUSP: domain present in USPs
TGF: transforming growth factor
FL: full-length
CD: catalytic domain
AMC: 7-amido-4-methylcoumarin
SL: switching loop
BL: blocking loop
PDB: Protein Data Bank
FAK: focal adhesion kinase
CCL: catalytic cleft loop.
