# Supplementary material for: The structure of the deubiquitinase USP15 reveals a misaligned catalytic triad and an open ubiquitin-binding channel
Source: J Biol Chem. 2018 Sep 18;293(45):17362–74. doi: 10.1074/jbc.RA118.003857 (PMC6231127; doi:10.1074/jbc.RA118.003857)
Supplement: Supporting Information [file supp_293_45_17362__index.html]

The structure of the deubiquitinase USP15 reveals a misaligned catalytic triad and an open ubiquitin-binding channel — USP15 catalytic domain structure — The structure of the deubiquitinase USP15 reveals a misaligned catalytic triad and an open ubiquitin-binding channel — USP15 catalytic domain structure — Supporting Information 

# The structure of the deubiquitinase USP15 reveals a misaligned catalytic triad and an open ubiquitin-binding channel

## Supporting Information

- Supporting Figures - Figure S1: Structure-based sequence alignment; Figure S2: Binding data of USP15-D1D2 and SL mutant USP15-D1D2 Cys352Ser with monoubiquitin; Figure S3: Diubiquitin cleavage assay gels; Figure S4: Superposition of USP15 structure with selected USP-ubiquitin complex structures
