# Supplementary material for: The structure of the deubiquitinase USP15 reveals a misaligned catalytic triad and an open ubiquitin-binding channel
Source: J Biol Chem. 2018 Sep 18;293(45):17362–74. doi: 10.1074/jbc.RA118.003857 (PMC6231127; doi:10.1074/jbc.RA118.003857)
Supplement: Supporting Information [file supp_RA118.003857_137987_2_supp_204026_pf7cdt.pdf]

SUPPORTING INFORMATION

The structure of the deubiquitinase USP15 reveals a misaligned catalytic triad and an open ubiquitin-binding channel

Stephanie J. Ward, Hayley E. Gratton, Peni Indrayudha[#], Camille Michavila, Rishov Mukhopadhyay, Sigrun K. Maurer, Simon G. Caulton, Jonas Emsley and Ingrid Dreveny*

From the Centre for Biomolecular Sciences, School of Pharmacy, University of Nottingham, Nottingham NG7 2RD, United Kingdom

Running title: *USP15 catalytic domain structure*

[#]Present address: Faculty of Pharmacy, Universitas Muhammadiyah Surakarta, Jl A.Yani Tromol Pos I, Pabelan, Kartasura, Sukoharjo, Indonesia 57102.

*To whom correspondence should be addressed: Ingrid Dreveny: Centre for Biomolecular Sciences, School of Pharmacy, University of Nottingham, Nottingham NG7 2RD, UK; ingrid.dreveny@nottingham.ac.uk; Tel.+44 1158468015; Fax. +44 01158468002.

Contents:

Supporting Figures:

- Figure S1
- Figure S2
- Figure S3
- Figure S4

Supporting references

Figure S1

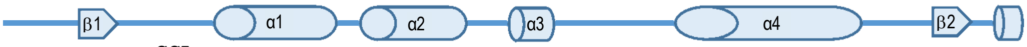


```
                               CCL
USP15A        MEQPGLCGLSNLGNTCFMNSAIQCLSNTPPLTEYFLNDKYQEELNFDNPLGMRGEIAKSYAELIKQMWSGKFSYVTPRAF 333
2Y6E_USP4A    -IQPGLCGLGNLGNTCFMNSALQCLSNTAPLTDYFLKDEYEAEINRDNPLGMKGEIAEAYAELIKQMWSGRDAHVAPRMF
2GFO_USP8A    -SGPALTGLRNLGNTCYMNSILQCLCNAPHLADYFNRNCYQDDINRSNLLGHKGEVAEEFGIIMKALWTGQYRYISPKDF
5k16_USP12A   ----HYFGLVNFGNTCYCNSVLQALYFCRPFREKVLAYKSQKKES---LLTCLADLFHSIATQKKKVG-----VIPPKKF
4m5x_USP7A    -KHTGYVGLKNQGATCYMNSLLQTLFFTNQLRKAVYMMPTEGDDSSKSVPLALQRVFYELQHSDKPVG--------TKKL
                     ** * * **: ** :* *     : . .     : . .          :        * :         .: :
Prim.cons.    M4QPGLCGL5NLGNTCYMNSALQCLSNTPPL22YFL5DKYQ52INRDN2LGM2GE2AE2YAELIKQ2WSG333Y24PKKF
```

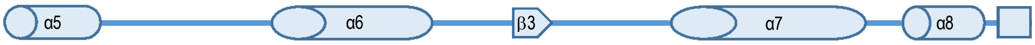


```
                              SL
USP15pdbA     KTQVGRFAPQFSGYQQQDCQELLAFLLDGLHEDLNRIRKKPYIQLKDADGRPDKVVAEEAWENHLKRNDSIIVDIFHGLF 413
2Y6E_USP4A    KTQVGRFAPQFSGYQQQDSQELLAFLLDGLHEDLNRVKKKPYLELKDANGRPDAVVAKEAWENHRLRNDSVIVDTFHGLF
2GFO_USP8A    KITIGKINDQFAGYSQQDSQELLLFLMDGLHEDLNKA---------DNDHLDDFKAAEHAWQKHKQLNESIIVALFQGQF
5k16_USP12A   ITRLRKENELFDNYMQQDAHEFLNYLLNTIADILQEE-----------RK-----------QEKQNPDPTWVHEIFQGTL
4m5x_USP7A    TKSFG--WETLDSFMQHDVQELCRVLLDNVENKMKGT----------------------------CVEGTIPKLFRGKM
                 .      : .: *:* :*:   *:: : : ::                                    :   *:* :
Prim.cons.    KTQVG2F22QF2GY2QQDSQELLAFLLDGLHEDLNR52KKPY22LKDADGRPD3VVAEEAW2NH44RN2SIIVD2F2GLF
```

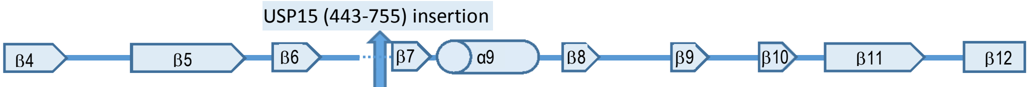


```
USP15A        KSTLVCPECAKISVTFDPFCYLTLPLPMK FVKLKDCIELFTTKEKLGAEDPWYCPNCKEHQQATKKLDLWSLPPVLVVH 805
2Y6E_USP4A    KSTLVCPECAKVSVTFDPFCYLTLPLPLK TVALRDCIELFTTMETLG-EDPWYCPNCKKHQQATKKFDLWSLPKILVVH
2GFO_USP8A    KSTVQCLTCHKKSRTFEAFMYLSLPLASTSKCTLQDCLRLFSKEEKLTDNNRFYCSHCRARRDSLKKIEIWKLPPVLLVH
5k16_USP12A   TNETRCLTCETISSKDEDFLDLSVDVE--QNTSITHCLRGFSNTETKY-----YCEECRSKQEAHKRMKVKKLPMILALH
4m5x_USP7A    VSYIQCKEVDYRSDRREDYYDIQLSIK--GKKNIFESFVDYVAVEQLDGDNK-YDAGEHGLQEAEKGVKFLTLPPVLHLQ
               .   *      *   : :  : : :       : ..:  :   *        *    :  ::: * ... .** :* ::
Prim.cons.    KSTL2C2ECAKISVTFE2FCYL2LPLPS3KKV5L5DC22LF2T5E2LG3E2PWYCPNC25HQ2ATKK52LW2LPPVLVVH
```

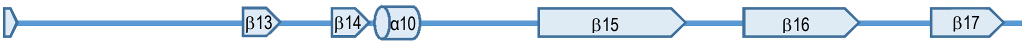


```
                    BL1                                              BL2
USP15A        LKRFSYSRYMR--DKLDTLVDFPINDLDMSEFLINPN-AGPCRYNLIAVSNHYG-GMGGHYTAFAKNKDDGKWYYFDDS 881
2Y6E_USP4A    LKRFSYNRYWR--DKLDTVVEFPIRGLNMSEFVCNLS-ARPYVYDLIAVSNHYG-AMGVGHYTAYAKNKLNGKWYYFDDS
2GFO_USP8A    LKRFSYDGRWK--QKLQTSVDFPLENLDLSQYVIGPK-NNLKKYNLFSVSNHYG-GLDGGHYTAYCKNAARQRWFKFDDH
5k16_USP12A   LKRFKYMDQLHRYTKLSYRVVFPLE-LRLFNTSGDAT-NPDRMYDLVAVVVHCGSGPNRGHYIAIVKS--HDFWLLFDDD
4m5x_USP7A    LMRFMYDPQTDQNIKINDRFEFPEQ-LPLDEFLQKTDPKDPANYILHAVLVHSG-DNHGGHYVVYLNPKGDGKWCKFDDD
              * ** *        *:.  . ** . * : :            * * :*  * *     *** .  :      *  ***
Prim.cons.    LKRFSYDR2WR22DKLDTRV2FP2E3LDLSEF2INP5P25P55Y2LIAVSNHYGSGMGGGHYTAYAKNK4DGKWY2FDD2
```

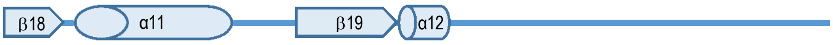


```
USP15A        SVSTASE---------DQIVSKAAYVLFYQRQDTFSGTGFFPL---------------------- 915
2Y6E_USP4A    NVSLASE---------DQIVTKAAYVLFYQRRDD-------------------------------
2GFO_USP8A    EVSDISV---------SSVKSSAAYILFYTSL---------------------------------
5k16_USP12A   IVEKIDAQAIEEFYGLTSKNSESGYILFYQSRD--------------------------------
4m5x_USP7A    VVSRCTKEEAIEHN-YGGHHCTNAYMLVYIRESKLSEVLQAVTDHDIPQQLVERLQEEKRIEAQK
               *.                    .*:*.*
Prim.cons.    5VS52SE2222E22G2D2IVSKAAY2LFYQRRD32S2222222DHDIPQQLVERLQEEKRIEAQK
```

Figure S1. Structure-based sequence alignment of selected USP catalytic core structures. Structure based sequence alignment of USP15, USP4 (PDB code: 2Y6E (1)), USP8 (PDB code: 2GFO (2), USP12 (PDB code: 5K16 (3)) and USP7 (PDB code: 4M5X (4)) with secondary structure elements as seen in the USP15 catalytic core structure indicated and numbered. Catalytic residues are highlighted in green, loop regions indicated (catalytic cleft loop: CCL; switching loop: SL; blocking loop 1: BL1; blocking loop 2: BL2) with residues mutated for ubiquitin binding experiments highlighted in yellow, and residues interacting with mitoxantrone within a 4Å radius of USP15, highlighted in orange underneath. Consensus symbols denote fully conserved residues (*), residues with strongly similar properties (:), residues with weakly similar properties (.), and different residues (unmarked).

Figure S2

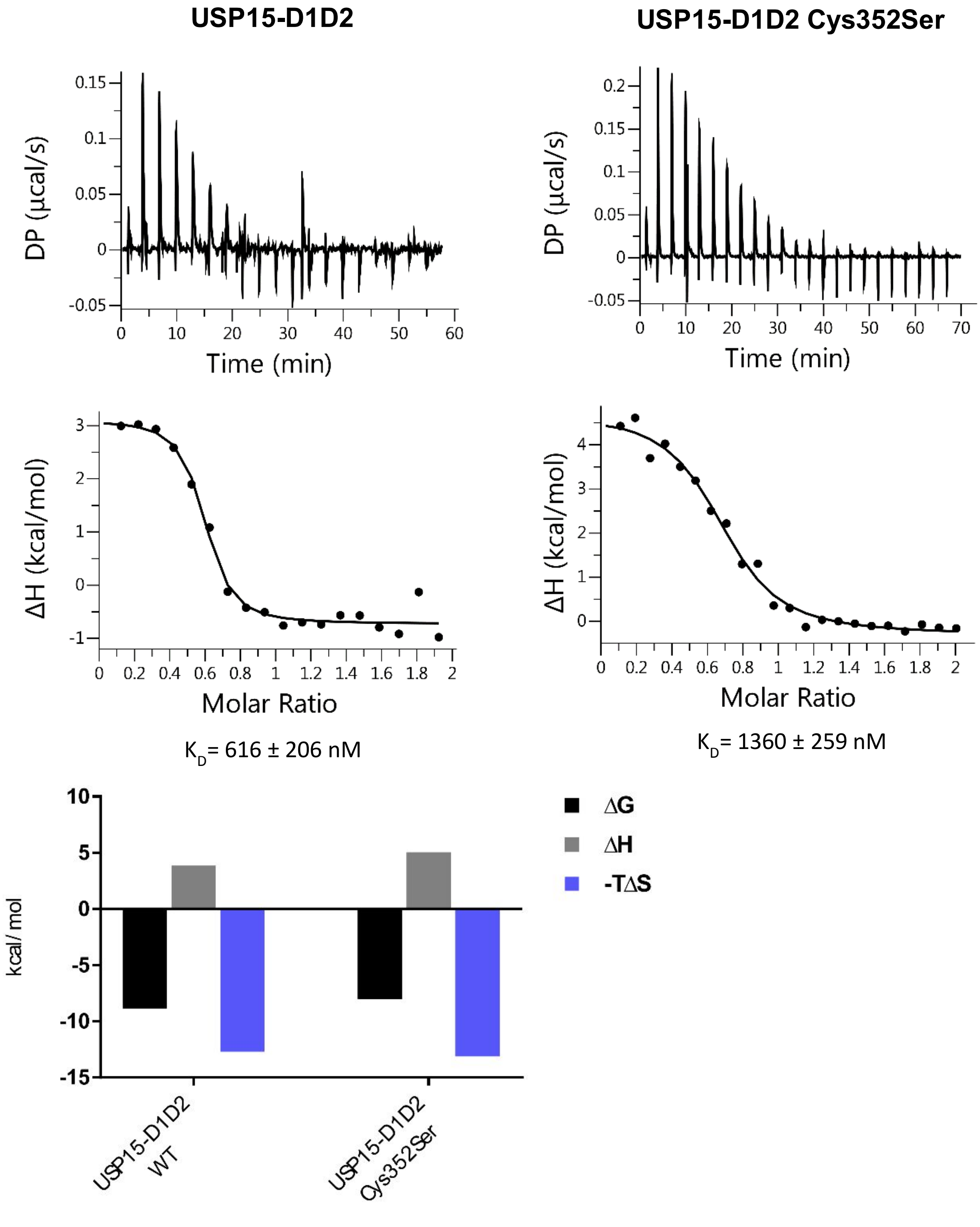

Figure S2. Comparison of binding interactions of USP15-D1D2 and SL mutant USP15-D1D2 Cys352Ser with monoubiquitin. ITC raw data and associated isotherms for USP15-D1D2 (left) and USP15-D1D2 Cys352Ser (right) titrations with monoubiquitin at 25°C with dissociation constants displayed below the graphs. A graphical representation shows that similar thermodynamic parameters of the interaction with ubiquitin are obtained upon this substitution in the USP15 SL rendering it identical to the SL sequence in USP4.

Figure S3

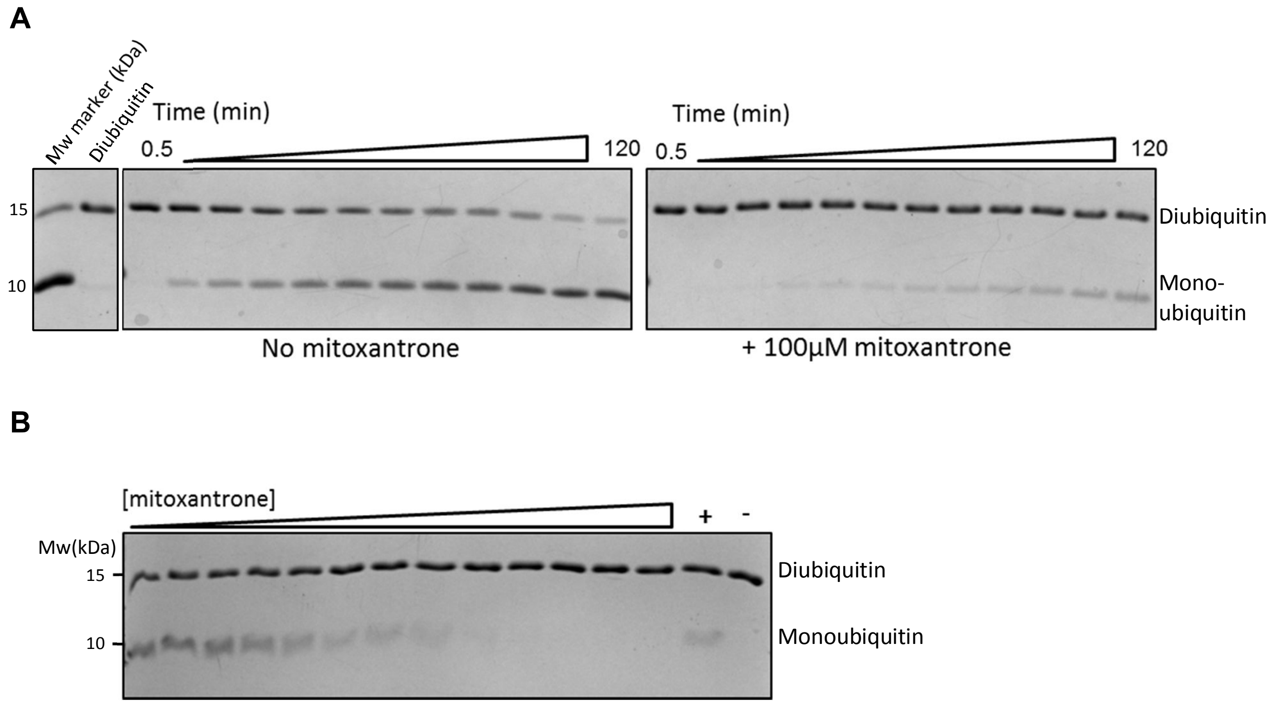

Figure S3. Diubiquitin cleavage assays. (A) Representative gels for diubiquitin cleavage assays using 400nM FL-USP15 and 5µM of linear diubiquitin as substrate at 25˚C in 50mM Tris-Cl pH 7.5, 300mM NaCl, 1% glycerol and 1mM DTT. Initial time course experiments were conducted over 2h with samples taken every 6min for 1h then at 90min and 120min; left: time course in the absence of mitoxantrone; right: time course in the presence of 100µM of mitoxantrone (B) Representative gel for diubiquitin cleavage assays under the conditions stated in (A) stopped after 30min in the presence of increasing concentrations of mitoxantrone in the range of 0.5-800µM. Positive (+) and negative (-) controls, consisting of untreated and denatured FL-USP15, respectively in the absence of mitoxantrone, are shown on the right of the gel.

Figure S4

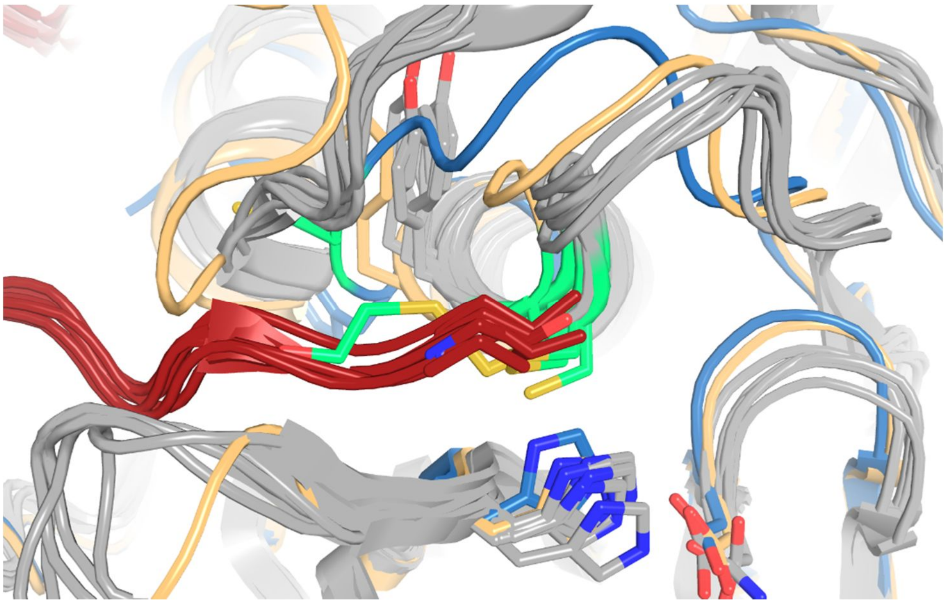

Figure S4. Superposition of USP15 catalytic core structure with selected USP-ubiquitin complex structures and USP4-BME. Structures of ubiquitin bound forms of USP2 (PDB ID: 2IBI), USP7 (PDB ID: 1NBF), USP21 (PDB ID: 3I3T), USP12 (PDB ID: 5L8W), USP14 (PDB ID: 2AYO), are shown in gray cartoon representation. USP15 and BME bound USP4 (PDB ID: 2Y6E) are coloured in blue and wheat, respectively. Ubiquitin molecules are depicted in red, active site cysteines in modified or unmodified form in green. Catalytic triad residues and CC loop aromatic residues following the catalytic cysteine are shown in stick representation.

References

1. Clerici, M., Luna-Vargas, M. P., Faesen, A. C., and Sixma, T. K. (2014) The DUSP-Ubl domain of USP4 enhances its catalytic efficiency by promoting ubiquitin exchange. *Nature communications* **5**, 5399
2. Avvakumov, G. V., Walker, J. R., Xue, S., Finerty, P. J., Jr., Mackenzie, F., Newman, E. M., and Dhe-Paganon, S. (2006) Amino-terminal dimerization, NRDP1-rhodanese interaction, and inhibited catalytic domain conformation of the ubiquitin-specific protease 8 (USP8). *J Biol Chem* **281**, 38061-38070
3. Li, H., Lim, K. S., Kim, H., Hinds, T. R., Jo, U., Mao, H., Weller, C. E., Sun, J., Chatterjee, C., D'Andrea, A. D., and Zheng, N. (2016) Allosteric Activation of Ubiquitin-Specific Proteases by beta-Propeller Proteins UAF1 and WDR20. *Mol Cell* **63**, 249-260
4. Molland, K., Zhou, Q., and Mesecar, A. D. (2014) A 2.2 A resolution structure of the USP7 catalytic domain in a new space group elaborates upon structural rearrangements resulting from ubiquitin binding. *Acta Crystallogr F Struct Biol Commun* **70**, 283-287
